# Liquid dynamic medicine and N-of-1 clinical trials: a change of perspective in oncology research

**DOI:** 10.1186/s13046-017-0598-x

**Published:** 2017-09-13

**Authors:** Nicola Silvestris, Gennaro Ciliberto, Paolo De Paoli, Giovanni Apolone, Maria Luisa Lavitrano, Marco A. Pierotti, Giorgio Stanta, On the behalf of the “dynamic medicine OECI group”

**Affiliations:** 1 Medical Oncology Unit and Scientific Directorate, Cancer Institute “Giovanni Paolo II”, Viale Orazio Flacco, 65, 70124 Bari, Italy; 2 Scientific Directorate, IRCCS National Cancer Institute “Regina Elena”, Rome, Italy; 3 Scientific Directorate, IRCCS “Centro di Riferimento Oncologico”, Aviano, Italy; 4 Scientific Directorate, Fondazione IRCCS Istituto Nazionale dei Tumori, Milan, Italy; 5 BBMRI.it and Department of Medicine and Surgery University Milano-Bicocca, Milan, Italy; 6 Senior Group Leader Foundation Institute FIRC Molecular Oncology (IFOM) Milan, Milan, Italy; 7 Department of Medical Sciences of the University of Trieste, Trieste, Italy

**Keywords:** Dynamic, Fluid, Personalized medicine, Target therapy, Clinical trials, N-of-1 trials

## Abstract

The increasing use of genomics to define the pattern of actionable mutations and to test and validate new therapies for individual cancer patients, and the growing application of liquid biopsy to dynamically track tumor evolution and to adapt molecularly targeted therapy according to the emergence of tumor clonal variants is shaping modern medical oncology., In order to better describe this new therapeutic paradigm we propose the term “Liquid dynamic medicine” in the place of “Personalized or Precision medicine”. Clinical validation of the “Liquid dynamic medicine” approach is best captured by N-of-1 trials where each patient acts as tester and control of truly personalized therapies.

## Background

In year 1999, Langreth and Waldholz used for the first time used the terminology “personalized medicine” with the aim indeed to identify “the right drug for each unique genetic profile” [1]. Since then, the expressions “personalized medicine”, or “precision medicine” (PM) have become widely used in oncology to indicate medical procedures by which patients receive tailored interventions based on the genetic alterations found in their tumors [2]. Although attempts to attain a unique definition of PM have been made, various definitions now exist in the literature which share the common concept that a genetic test is at the basis of every PM treatment and that this genetic test is required to stratify patients into subgroups which may or may not take advantage from a medical treatment [3, 4]. It is in this context that PM is strictly based on a molecular investigation. However, this does not necessarily correspond to personalization of the care tailored to the patients’ preferences and choices, which often is the cause of confusion [5]. Hence, today the term personalized or precision medicine is interpreted in various ways by the media, health care professionals or patients [6]. Perhaps the most appropriate definition, because it is the most widely used, is the National Institutes of Health’s definition: “The use of the combined knowledge (genetic or otherwise) about a person to predict disease susceptibility, disease prognosis, or treatment response and thereby improve a person’s health” [6].

## Genomic biomarker-driven therapy

Holding onto the concept of PM as a genetic biomarker-driven treatment, an increasing number of cases have been accumulated in recent years. Of these, the most cited examples include the use of imatinib for BCR-ABL positive chronic myeloid leukemia or c-Kit positive gastrointestinal tumors [7], anti-HER2 antibodies for HER2 amplified breast or gastric cancers [8], anti-EGFR antibodies for non KRAS mutated colorectal cancers [9], small molecule EGFR inhibitors for EGFR mutated lung cancers [10, 11], BRAF inhibitors or combinations of BRAF and MEK inhibitors for melanoma patients bearing BRAF mutations in their tumors [12] or ALK inhibitors for ALK or ROS translocations in lung cancer [13]. Conventional Phase III trials have shown that when patients are stratified by the use of a genetic test known as “companion diagnostics” to receive targeted therapy, patients testing “positive” for the biomarker experience a superior clinical benefit in terms of progression free survival and/or overall survival as compared to those testing “negative” for the same biomarker. This approach has often allowed accelerated market approval for the corresponding drugs [14]. It must be added that meta-analyses including a total of approximately 85,000 patients have confirmed that the genetic biomarker-driven patient selection is safe and has been associated with improvements in all outcome variables [15–17]. In addition, it would be considerate that the increasing use of this molecular approach in both cancer research and clinical practice, bring to a higher expense for target drugs, which are compensated by a less overall costs for the Health System coming from the better patient outcome and reduction of hospital admissions.

However, strictly speaking, these examples of PM are not truly considered “personalized medicine” since they are not tailored to individual patients, but rather to subgroups of patients sharing only one of the several genetic alterations present in their tumors.

The genetic biomarker-driven concept of PM has been challenged by a series of facts and evidence. Firstly, the presence or absence of the specific biomarker does not always result in biological and clinical sensitivity to the corresponding drug. For example, a subset of lung cancer patients which do not bear activating EGFR mutations can achieve clinical responses to EGFR inhibitors [18], or also a good proportion of BRAF mutated melanoma patients do not respond to BRAF inhibitors [19]. The case of BRAF mutations is even more intriguing because activating oncogenic BRAF mutations are found in several other tumors, including colorectal, thyroid, lung cancer but in the majority of those cases they are not predictive of drug response to the same BRAF inhibitors as in melanoma. Mechanistic explanations to these findings are emerging and reside in the presence of additional genetic or epigenetic alterations which may create from case to case “favorable” or “unfavorable” contexts to the action of a specific target therapy. This brings us to the second line of evidence: tumors are in general highly heterogeneous and mutated in several driver genes.

## Interpatient and intrapatient heterogeneity

The first level of heterogeneity is interpatient heterogeneity. Tumors of the same histological origin but deriving from different patients, are genetically (and epigenetically) altered and harbor a large number of molecular alterations resulting from an evolutionary process that starts from normal cells through the clonal expansion of cells capable of overcoming the physiological control of cell growth. At this point the necessity, for true “precision oncology”, is to identify all molecular alterations (genomic or not) of cancer that can shape response to treatments [20]. Increased optimism in recent years has been generated by technical improvements and decreased costs of next generation sequencing (NGS). It is now possible, at least in theory, to use gene panels of increasing complexity to identify all driver genetic mutations by NGS and match these mutations to an ever increasing number of approved or experimental drugs capable of targeting these mutations [21, 22]. Applying of this concept is a highly challenging task because of the complexity to accumulate, store, interpret and standardize the data required to leverage genomic data that improves patient treatment [23]. However, this is not yet feasible in clinical practice at the present time and only few organizations have been able to use this approach experimentally with encouraging results [21, 22]. In addition to a highly qualified bioinformatic capable of elaborating data, it is also necessary to assemble and coordinate of a multidisciplinary tumor board comprising oncologists, radiologists, pathologists, geneticists, statisticians, mathematicians, as well as partnering up with several pharmaceutical companies to make their experimental drugs available. Hence, with a few exceptions, although we are able to identify several genomic aberrations in metastatic cancer, the utility of this information still remains largely elusive [23].

Therefore, at this moment in time we cannot talk of the realization of a true “precision medicine” approach. This may be also due to an additional reason, namely the intrapatient heterogeneity of tumors and the ability of cancer genomes to evolve dynamically over time and accumulate different subsets of mutations in different tumor subclones. Sophisticated techniques are able to construct phylogenetic trees of tumors showing the relationships among the various patterns of mutations [24]. Different subclones can change in their relative proportions with time due to selective pressures, endogenous (e.g. immunosurveillance by our immune system), or exogenous (environmental cues or drug treatments). Nowadays more than ever, we can observe as a result of the application of sequential lines of therapy often lasting years in the same metastatic patient, that cancer is becoming a chronic disease. The notion of chronicity means that cancer is continuously evolving and is genetically very different after years of therapy far from the time of the initial diagnosis.

## From “personalized or precision medicine” to “liquid dynamic medicine”

One of the most important breakthroughs in the last few years is that it is now technically possible to follow tumor evolution in a non-invasive manner by a procedure called “liquid biopsy” which involves sequencing tumor DNA fragments known as circulating tumor DNA (ctDNA) in blood samples [25]. Liquid biopsies are already in use in selected cases for diagnostic purposes (for example the detection of resistant mutations in EGFR), and we can expect a dramatic rise of clinical applications, given that they can predict disease relapse several weeks before radiological detection of disease recurrence [26]. Hence, we can expect future cancer therapies to not only be genomically driven but also continuously determined by the variations provided by the results of sequential liquid biopsies capable of tracking changes of the emerging dominant subclones to be targeted by the increasing number of matching drugs [27]. On this basis, and according to Bauman’s definition of “liquid-modern society” [28] we would like to propose to replace the expression “personalized or precision medicine” to “liquid dynamic medicine” which better describes this methodological approach. “Liquid dynamic medicine” accurately describes dynamic changes in tumor evolution which imposes dynamic changes in the therapy to apply, but also based on the fact that relevant information is present in body fluids. In the “liquid dynamic medicine” scenario patients (and their associations) play a central role and are key players as the information for therapy directly derives from their blood or other body fluids, to which they are the sole contributors. Also, central to realizing of this scenario is the capability of building and interrogating biobanks of longitudinal body fluid samples [29]. Moreover we believe that the “liquid dynamic medicine” approach will apply to the design and construction of patient-specific cancer vaccines against neoantigens a new fashionable approach to cancer therapy [30].

## N-of-1 trials as a tool to implement “liquid dynamic medicine”

Toward the practical realization of this “liquid dynamic medicine” setting the use of unconventional clinical trials is required. Conventional phase I-III clinical trials along with their rigid schemes do fail to respond the need of answering more questions, more efficiently and in less time. They are unable to respond to the need of a dynamic therapy in which the biological features of the disease change with time and also where every patient, due to the growing complexity of using diagnostic testing will be differentiated from all the others. One attempt to solve this issue is by the use of the so called “Master Protocols”, designed to answer multiple questions [31]. PM’s master protocol concept challenges the traditional clinical trial infrastructure through evaluating more than one or two treatments in more than one patient type or disease within the same overall trial structure. Master protocols include umbrella, basket and platform trials. Umbrella trials are designed to study targeted therapies in the context of a single disease. Basket trials study a single targeted therapy in the context of multiple diseases or a disease subtype. Platform trials focus on multiple targeted therapies in the context of a single disease in a perpetual manner, allowing therapies to enter or leave the platform according to a decision algorithm.

**Fig. 1:**
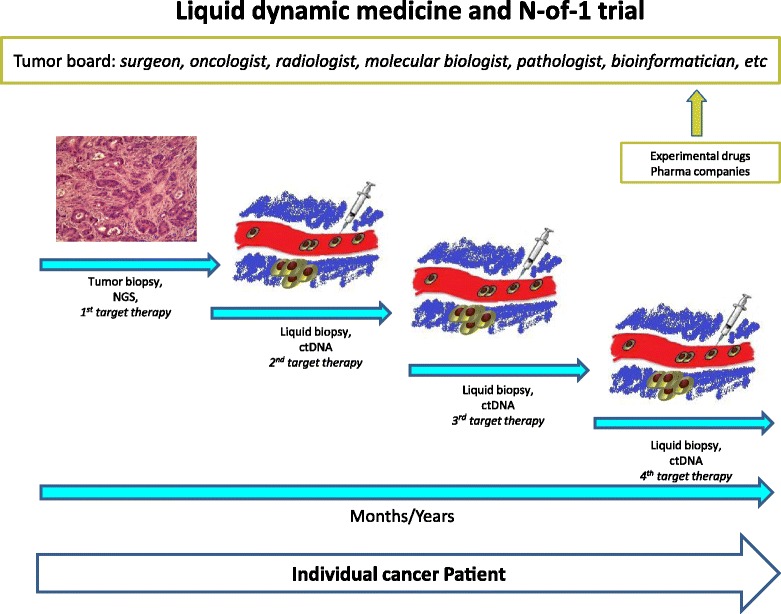
Liquid dynamic medicine and N-of-1 trial

We believe however that also Master Protocols are going to reveal their limitations in the new world of “liquid dynamic medicine” and extreme personalization of care. Indeed, in cases where a combination of molecular alterations is very rare, testing the activity and efficacy of investigational drugs in a sufficient number of patients also within master protocols will be highly challenging. For example, while 5% of patients with a common malignancy (e.g., breast cancer) may be sufficient to conceive and conduct a conventional trial of a new target therapy, enrolling an adequate number of patients in a timely manner to define clinical utility would be extremely difficult if the population in question represented 1% or less of this population, and virtually impossible if one wishes to explore the benefits of treatment in rarer neoplasms. However, this goal could be possible to achieve with studies focusing on a single person – known as N-of-1 trials – which aim to study targeted treatments for tumors in individual patients (Fig. 1) [32]. The option we propose is to compare the time-to-disease progression of an individual cancer patient following treatment with a novel therapy to the time-to-disease progression for the same patient on his/her immediately previous treatment [33]. In other words, in N-of-1 trials the same patient will be the tester of a new therapy and its control arm.

## Conclusion

The increasing knowledge of molecular events in cancer cells prompted the identification of targeted therapies which could be able to interfere with tumor growth. A dynamic chess mate appeared to characterize sequential molecular events developing between the onset of mechanisms of resistance and the identification of new therapeutic strategies.

In conclusion, therefore, for testing new oncology therapies based on in depth genomic characterization of patient’ tumors, we propose the use of N-of-1 trials and the promotion of the term “liquid dynamic medicine”.
